# Intra-osseous Ice Ball Visualization During Cryoablation Using Photon-Counting Detector Computed Tomography: An In-Vivo Swine Pilot Study

**DOI:** 10.1007/s00270-025-04343-w

**Published:** 2026-02-08

**Authors:** Sivert Kupfer, Konstantin Klambauer, Gilbert Puippe, Miriam Weisskopf, Thomas Pfammatter, Thomas Flohr, Adrian Kobe

**Affiliations:** 1 Institute of Diagnostic and Interventional Radiology, University Hospital Zurich, Zurich, Switzerland; 2 Center for Surgical Research, University of Zurich, University Hospital of Zurich, Zurich, Switzerland; 3 Department of Radiology and Nuclear Medicine, Maastricht University Medical Centre+, Maastricht, The Netherlands

**Keywords:** Cryoablation, Dual source photon counting detector CT, Virtual non calcium images, Ice ball

## Abstract

**Purpose:**

To evaluate the visualization of the ice ball during cryoablation of vertebral bodies and the bony pelvis in an in vivo swine model using virtual non-calcium imaging (VNCa) derived from photon-counting detector computed tomography (PCD-CT).

**Materials and Methods:**

Cryoablation was performed at six locations in the spine and pelvis of three live swine. Spectral ultra-high resolution image acquisition using PCD-CT was conducted before ablation and at 2, 4, 6 and 8 min after initiation of the cryoablation cycle. Artifacts were suppressed using iterative metal artifacts reduction. Ice ball visualization within bone and soft tissue was assessed on both, conventional and VNCa images. At each time point the presence and size of the ice ball visualization was evaluated.

**Results:**

A total of 12 needle placements was analyzed. In conventional images the ice ball could not be seen within bone at any time. In contrast, in VNCa images the ice ball became visible after 2 min in all cases. However, the ice ball was less clearly visualized in the soft tissue on VNCa images. Mean ice ball volume at 2, 4, 6, 8 min was 1.6 ± 0.9, 2.8 ± 1.7, 5.5 ± 2.9 and 8.3 ± 3.2 ml in the vertebral bodies and 0.8 ± 0.3, 2.0 ± 0.4, 4.4 ± 0.6, 6.2 ± 1.1 ml in the bony pelvis, respectively.

**Conclusion:**

PCD-CT enables early visualization of the ice ball within vertebral bodies and the bony pelvis in a swine model using VNCa images.

**Graphical Abstract:**

**Figure:**
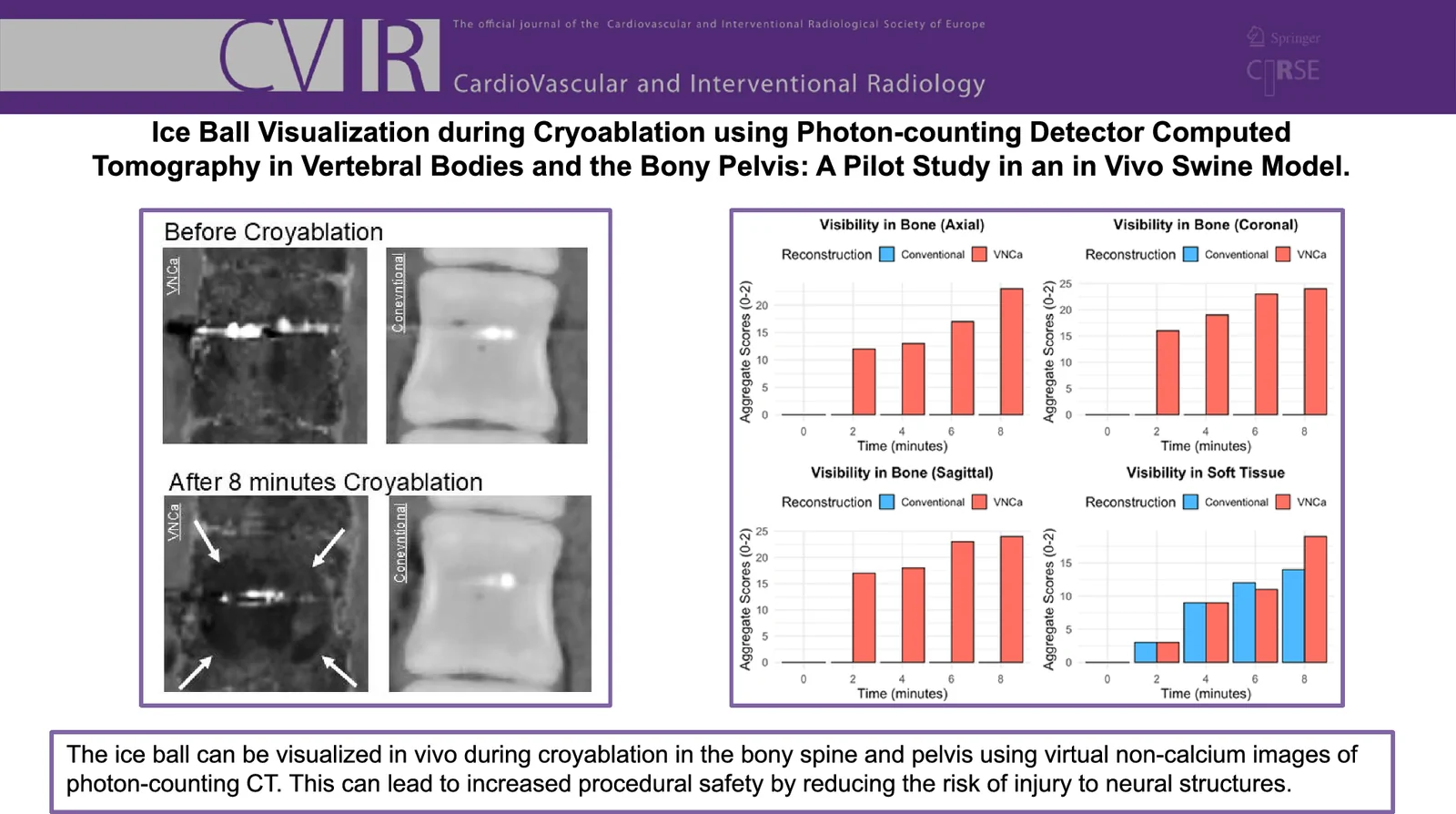

**Supplementary Information:**

The online version contains supplementary material available at https://doi.org/10.1007/s00270-025-04343-w.

## Introduction

Bone metastases are present in up to 85% of patients with cancer at the time of death. They are associated with substantial clinical morbidity, including impaired mobility, severe pain, and an elevated risk of pathological fractures. As a result, effective management of bone metastases is essential for maintaining and improving quality of life in cancer patients [1–3].

Since the first prospective study on percutaneous cryoablation of painful bone metastases in 2006, this palliative treatment has consistently been shown to be a safe and effective therapeutic option [3–6]. The major disadvantage, however, is that during the actual cryoablation procedure, the ice ball within bone cannot be visualized on conventional CT and becomes apparent only once it extends beyond the osseous boundary [7]. As a result, critical structures adjacent to the bone, such as the spinal cord or nerves may be at risk of injury [8, 9]. To mitigate this risk, a technique capable of visualizing the ice ball within bone in real time is required.

Morris et al. [10] were the first to attempt visualization of the ice ball within osseous structures in cadaveric models using dual-energy CT. Their study demonstrated that the ice ball could be visualized nearly in real time on virtual non-contrast generated images in both, the vertebral column and pelvic bone. One drawback of the study was that the results could not capture the influence of blood flow and its cold sink effect on the ice ball [11, 12].

Recently a novel CT technology—the photon-counting detector CT (PCD-CT)—has entered clinical practice. This technology is capable of counting individual X-ray photons and measuring their energy in pre-defined energy bins, representing a fundamental shift in CT imaging. Thanks to spectral data acquisition, virtual non-calcium (VNCa) images can be generated, in which calcium signals are subtracted.

The aim of the present study is to evaluate the feasibility of visualizing the ice ball during cryoablation of vertebral and pelvic bone in an in vivo swine model using VNCa images with a dual-source photon-counting detector CT (DS PCD-CT).

## Materials and Methods

### Animal Protocol

The reporting of this study adheres with the Animal Research: Reporting of In Vivo Experiments 2.0 guidelines; the checklist is provided in Supplemental Digital Content (Supplementary Data 1, http://links.lww.com/TA/D689) [13]. The animal housing and the study protocol were approved by the local Veterinary Office (ZH164/2023) and were in accordance with National Animal Protection Laws and the European Directive 2010/63/EU of the European Parliament and of the Council on the Protection of Animals used for Scientific Purposes.

A total of 3 female animals (Swiss large white race, 65 ± 5 kg) were included in this study.

At the beginning of the experiment, all animals received premedication with ketamine (20 mg/kg), azaperone (2 mg/kg), and atropine (0.05 mg/kg) intramuscularly. After loss of postural reflexes, the anesthesia was deepened by a bolus injection of propofol (1–2 mg/kg) to facilitate intubation. Anesthesia was maintained with 2–3% isoflurane and propofol (2–5 mg/kg/h) for the remainder of the experiment. Pain management included fentanyl infusion (0.02 mg/kg/h) for the duration of the procedure.

On the completion of the study procedure (4–5 h long), all animals were euthanized by the administration of an overdose of pentobarbital while still under general anesthesia according to the animal study protocol.

### Cryoprobe Placement and Ablation Protocol

For cryoablation, four vertebral bodies (L 2–5) and the two respective iliac wings were selected in each of the three pigs. The animals were positioned prone. Two trained interventional radiologists, one with five years of experience in interventional radiology and one with one year of experience, performed all procedures.

In order to guide the cryoprobes safely to the target site, the first step was to insert biopsy needles (Bonopty® Penetration Set, 14G) under CT guidance (SOMATOM Edge plus, Siemens Healthineers AG) using a transpedicular access to the vertebral bodies and in the iliac wing from the dorsal side via the iliac crest in the axis of the iliac wing within bone marrow.

Two types of 17G cryoprobes were inserted consecutively over the biopsy needles in coaxial technique. IceSphere™ (VISUAL ICE™, Boston Scientific, Natick, MA) were inserted into lumbar vertebral bodies 2 to 4 and IceRod™ (VISUAL ICE™, Boston Scientific, Natick, MA) were inserted into lumbar vertebral body 5 and the iliac bone, respectively. The biopsy needles were then retracted so that the tip of the cryoablation needle was not covered by the biopsy needle. An argon-based cryoablation unit (ICEFX; Galil Medical, Yokneam, Israel) was used to perform the cryoablation. To ensure consistency across all procedures, cryoablation parameters—including probe type, manufacturer settings (freezing at 100%), and the duration of freezing cycle (8 min)—were standardized and applied uniformly across all animals and target sites.

### CT Scan Protocol

Visualization of the ice ball was performed with a first-generation DS PCD-CT scanner (NAEOTOM Alpha, Siemens Healthineers AG). A scan was performed before the start of cryoablation. After the start of cryoablation, a scan was performed at time intervals of 2, 4, 6 and 8 min to visualize the ice ball. For each time interval, one scan was performed with an ultra-high-resolution (UHR) spectral mode with detector collimation of 120 × 0.2 mm, spiral pitch factor of 0.85, and gantry rotation time of 0.5 s. An X-ray tube voltage of 140 kV at an IQ-level of 127 was used with automatic tube current modulation (CAREDose4D, Siemens Healthineers AG). Spectral post-processing (SPP) images were reconstructed with 1.5 mm slice width, kernel Qr36 and quantum iterative reconstruction (QIR) level 3, with iterative metal artifact reduction (iMAR, Siemens Healthineers AG).

### Postprocessing

After image acquisition, the SPP images were imported into a commercial software platform for further processing (syngo VIA, Siemens Healthcare GmbH). From each acquired CT scan, at 0, 2, 4, 6 and 8 min, two different image series were reconstructed: conventional CT images and VNCa images. The conventional images were generated using virtual monoenergetic images (VMI) at 70 keV. In order to generate VNCa images, the application for virtual non-contrast imaging (syngo.CT DE Virtual Unenhanced) was used and the parameters were adjusted to subtract calcium instead of iodine contrast agent. The most important change was an adjustment of the “Iodine Ratio” to 1.56. Additionally, the parameter ‘Resolution’ was set to 3, and the ‘Maximum (HU)’ to 3071. The generated VNCa and conventional images were reconstructed in axial, coronal and sagittal planes for image analysis.

### Image Evaluation

For each location and time point, the two performing interventional radiologists independently assessed both, the conventional and VNCa images to determine whether the ice ball within the bone and/or adjacent soft tissue was subjectively recognizable. A three-point Likert scale was used (scale 0 (no visual ice ball), 1 (slightly visible ice ball) and 2 (clearly visible ice ball)). In addition, the size of the ice ball on the VNCa images at each location and time point was measured (anteroposterior [AP] × left to right [LR] x supero-inferior [SI]). The volume of the ice ball was calculated using a formula for ellipsoidal geometry: *V* = 4/3⋅*π*⋅AP/2⋅LR/2⋅SI/2. The interference of the metal artifacts on the visibility of the bone and soft tissues was assessed using a 4-point Likert scale of 0 (no artifacts, good visibility of bone and soft tissue), 1 (minimal artifacts, impaired visibility of bone and soft tissue), 2 (moderate artifacts, reduced visibility of bone and soft tissue) and 3 (severe artifacts, poor visibility of bone and soft tissue).

### Study Outcome Measure and Statistical Analysis

Data were categorized by time points (0, 2, 4, 6, 8 min), anatomical regions (L2-5 and iliac wing), measurement types (visibility and artifacts), and images (conventional and VNCa). Combined anatomical regions (lumbar vertebral body combined, iliac wing combined, all combined) were also analyzed to provide a broader overview. Given the exploratory nature and very small sample size, the analysis focused on descriptive statistics (mean ± SD, median, Q1–Q3). Comparisons between conventional and VNCa images were additionally explored using the Wilcoxon rank-sum test.

Time-series data visualization was conducted to observe trends over the different time points. Ordinal data, such as visibility scores or artifacts, were visualized using bar plots, while continuous data, such as volume were represented using line plots with data points to indicate mean changes over time. All tests used a significance level of < 0.05. Interreader and intrareader reliability for volume measurements was assessed with the intraclass correlation coefficient (ICC) and for visibility measurements with Cohen's Kappa (κ) coefficient. Intraclass correlation coefficient (ICC): Values less than 0.5 are indicative of poor reliability, values between 0.5 and 0.75 indicate moderate reliability, values between 0.75 and 0.9 indicate good reliability, and values greater than 0.90 indicate excellent reliability [14]. Cohen's kappa coefficient: 0.21–0.40 indicated fair, 0.41–0.60 moderate, 0.61–0.80 good, and a kappa > 0.81 indicated excellent agreement [15]. Statistical analyses were performed using open-source software (R, version 4.4.0, R Core Team, Vienna, Austria).

## Results

### Visibility and Volume of the Ice Ball

**Fig. 1:**
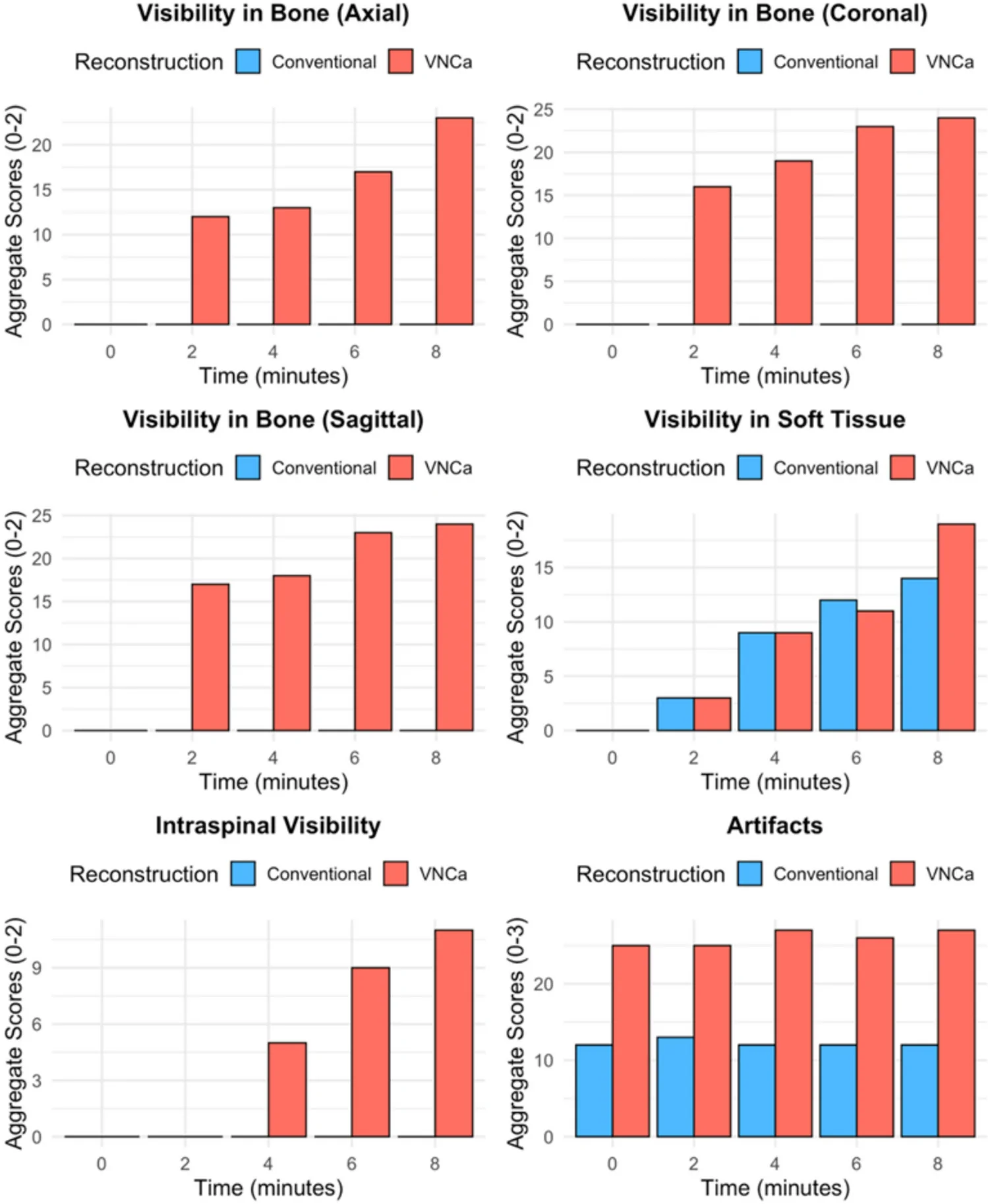
Bar charts show the visibility of the ice ball in bone and soft tissue in the conventional images and in the VNCa images, as well as the intraspinal visibility and artifacts

**Fig. 2:**
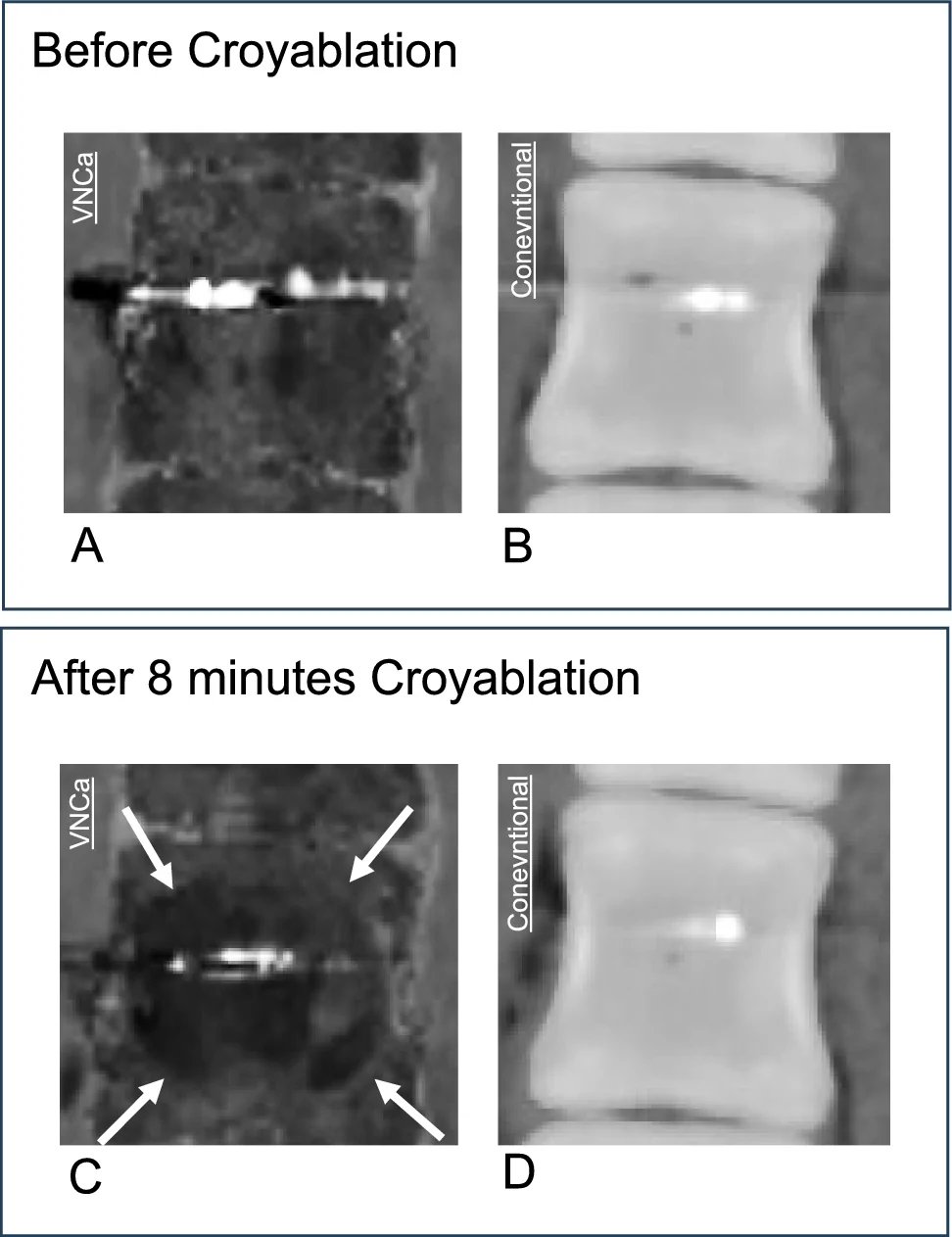
Ice ball formation in the spine before and after 8 min of freezing cycle. (marked with an arrow). **A** and **B** Images acquisition before Cryoablation with no ice ball visible. **C** and **D** after 8 min of freezing cycle with visible ice ball only in the VNCa images (**C**) and not in the conventional images (**D**). VNCa: **A** and **C**; Conventional: **B** and **D**

Figure 1 and supplementary Table 1 summarize the visibility of the ice ball across all anatomical sites in both, the bone and soft tissue, assessed in different imaging planes. The ice ball within the bone was not visible at any time on conventional images, but was consistently visualized on VNCa images (Table 1), representative CT images are shown in Fig. 2. On VNCa images, the ice ball became detectable in bone as early as 2 min after initiation. The best visualization in bone was achieved in the sagittal plane, followed by the coronal and axial planes (Table 2). In soft tissue, the ice ball was equally visible on both conventional and VNCa images at the 2-min time point. At 6 min, its visibility was partially reduced on VNCa images compared with conventional CT, whereas at 8 min, VNCa provided equal or improved visualization. Intraspinal extension of the ice ball was detectable only on VNCa images.

**Table 1:** Ice ball visibility (VNCa vs. conventional) at 6 and 8 min

Time (min)	Location	VNCa, median (IQR)	Conventional median (IQR)	*p*
6	All (bone + soft tissue)	2 (1–2)	0 (0–0)	< 0.001
6	Bone only	2 (1–2)	0 (0–0)	< 0.001
6	Soft tissue only	1.5 (1–2)	1.5 (1–2)	0.83
8	All (bone + soft tissue)	2 (1–2)	0 (0–0)	< 0.001
8	Bone only	2 (1–2)	0 (0–0)	< 0.001
8	Soft tissue only	2 (2–2)	1.5 (1–2)	0.024

**Table 2:** Ice ball visibility on VNCa images

Orientation	Sagittal	Coronal	Axial
Median (IQR)	2 (1–2)	1.5 (1–2)	1 (1–2)

Comparison	*p*
Sagittal vs. axial	< 0.001
Coronal vs. axial	< 0.001
Sagittal vs. coronal	0.874

**Fig. 3:**
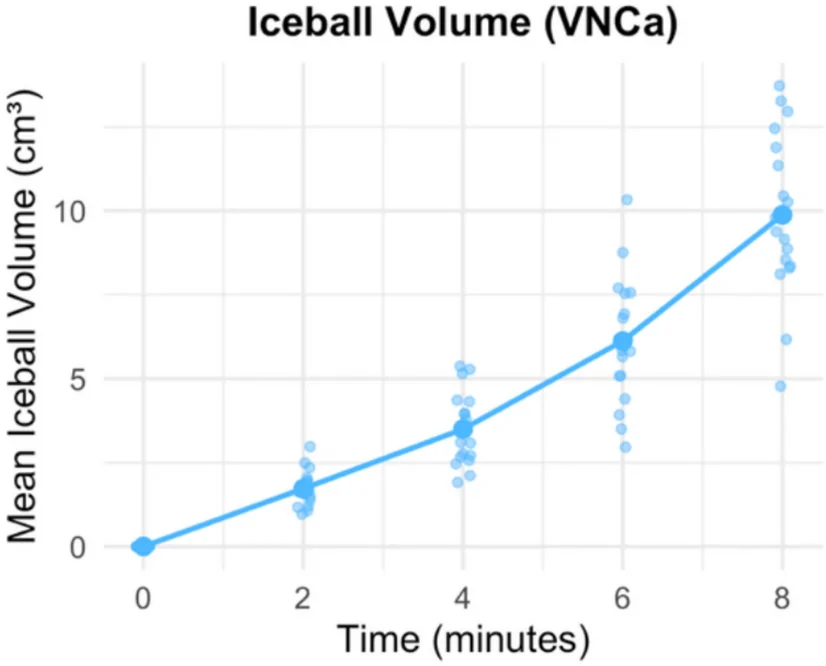
Graphical representation of ice ball volume over time

The ice ball volumes in the various locations and time points are summarized in Table 3, Fig. 3, and supplementary Table 1. The interreader agreement for the ice ball volume was excellent (ICC 0.95, *p* < 0.001) as well as for ice ball visibility (Cohen *κ*, 0.925, *p* < 0.001).

**Table 3:** Ice ball volume at different time points

Time (min)	Mean ± SD (cm^3^)
0	0 ± 0
2	1.72 ± 0.52
4	3.5 ± 1.08
6	6.12 ± 1.87
8	9.88 ± 2.43

### Metal Artifact

The metal artifacts were ubiquitous in all reconstructions, especially in the axial section plane with axial alignment along the cryo-probe. The metal artifacts were significantly more pronounced in the VNCa images than in the conventional images (Fig. 1).

## Discussion

The current study demonstrates that DS PCD-CT enables early visualization of the ice ball within vertebral bodies and the bony pelvis in an in-vivo swine model using virtual non contrast images. A currently unsolved problem in conventional CT guided bone cryoablations. Especially the high interreader agreement between the two radiologists for both, subjective visibility and the measured ice ball size, has to be highlighted.

However, there were two disadvantages of the qualitative evaluation of VNCa images, which were already described in the study by Morris et al. [10]: the increased metal artifacts on the one hand and the reduced visibility of the ice ball in the soft tissue on the other hand. The metal artifacts were particularly pronounced in the axial plane, along the axis of the cryoprobes, and were in some cases so extensive that assessment of the ice ball on VNCa in the axial plane was not feasible. In everyday clinical practice, however, the live generated and VNCa reconstructed images would be viewed in the plane of the gantry (*x*–*y* plane) where most artifacts occur. One solution would be simultaneous coronal and sagittal reconstruction of the VNCa image during ablation. Another concern with the VNCa images was the partially reduced visibility of the ice ball in the soft tissue, particularly after 6 min. This is probably due to pixelated image texture and high noise of the VNCa images and the artifacts [16].

Due to the substantial data volumes, another issue is the current lack of integration into the clinical workflow for real-time intraoperative feedback. The data must first be exported from the scanner to a software platform, and the parameters required to generate VNCa images must then be entered manually before the ice ball can be evaluated. Based on the reconstruction workflow during the experimental sessions, image reconstruction required approximately several minutes (on the order of ~ 10 min), which currently limits applicability in routine clinical settings. Reconstruction time was not prospectively measured as a predefined outcome parameter. A potential solution would be the implementation of an on-scanner software, enabling live visualization of VNCa images allowing hybrid-imaging-guided cryoablations. Real-time visualization of the ice ball may offer interventionalists greater confidence during bone ablation and could potentially allow more aggressive yet safe treatments.

A major limitation of this study is that the experiments were conducted in healthy swine bone. In clinical practice, cryoablation is typically performed in osteolytic, mixed, or sclerotic metastases, which differ in architecture and thermal conductivity. Future studies should therefore evaluate ice ball behavior and PCD-CT visibility across these pathological bone types. In addition, with only three live swine, the sample size was limited, and the results should be interpreted as preliminary. Validation in larger cohorts and across additional anatomical sites is required to confirm generalizability.

Another limitation is that ice ball visibility was assessed solely using a subjective Likert scale, without concurrent temperature measurements or histopathologic correlation. Furthermore, all procedures were performed using 17G cryoprobes, reflecting our clinical practice and experience. Larger-diameter probes (e.g., 14G) may produce more pronounced metal artifacts, which could adversely impact ice ball visualization on VNCa images.

In conclusion, this study demonstrates that the ice ball within osseous structures of the spine and pelvis can be visualized in a live swine model using VNCa images from a novel photon-counting CT. This capability may enhance procedural safety by reducing the risk of injury to neural structures. In the future, this technology could play an important role in cryoablation of osseous tumors in routine clinical practice.

## Supplementary Information

Below is the link to the electronic supplementary material.

Supplementary file1 (DOCX 18 KB)

## Abbreviations

DECT: Dual-energy CT
PCD-CT: Photon-counting detector CT
DS PCD-CT: Dual-source photon-counting detector CT
VNCa: Virtual non-calcium images
UHR: Ultra high resolution
