# Supplementary material for: Intra-osseous Ice Ball Visualization During Cryoablation Using Photon-Counting Detector Computed Tomography: An In-Vivo Swine Pilot Study
Source: Cardiovasc Intervent Radiol. 2026 Feb 8;49(9):1886–92. doi: 10.1007/s00270-025-04343-w (PMC13768354; doi:10.1007/s00270-025-04343-w)
Supplement: Supplementary file 1 [file 270_2025_4343_MOESM1_ESM.docx]

| Pig | Location | Cryoneedle | Time to Visualization Bone conventional | Time to Visualization tissue conventional | Time to Visualization Bone VNCa | Time to Visualization tissue VNCa | Ice Volume, ${cm}^{3}$, 8 min | Ice ball Dimension cm, 8 min |
| --- | --- | --- | --- | --- | --- | --- | --- | --- |
| 1 | L2 | IceSphere | none | 6 min | 2 min | 6 min | 8,9 | 3,95 x 2,2 x 1,95 |
| 1 | L3 | IceSphere | none | 8 min | 2 min | 8 min | 9,0 | 3,95 x 2,3 x 1,9 |
| 1 | L4 | IceSphere | none | 4 min | 2 min | 4 min | 8,7 | 3,55 x 2,4 x 1,95 |
| 1 | L5 | IceRod | none | 2 min | 2 min | 2 min | 12,9 | 4,6 x 2,5 x 2,15 |
| 1 | Iliac wing right | IceRod | none | 2 min | 2 min | 2 min | 11,1 | 5,05 x 2,15 x 1,95 |
| 1 | Iliac wing left | IceRod | none | 2 min | 2 min | 2 min | 11,8 | 4,95 x 2,1 x 2,15 |
| 2 | L2 | IceSphere | none | 8 min | 2 min | 8 min | 8,4 | 4,05 x 2,15 x 1,85 |
| 2 | L3 | IceSphere | none | 4 min | 2 min | 4 min | 5,5 | 3,35 x 1,95 x 1,6 |
| 2 | L4 | IceSphere | none | none | 2 min | none | 9,9 | 3,65 x 2.25 x 2.3 |
| 2 | L5 | IceRod | none | 2 min | 2 min | 2 min | 13,6 | 4,7 x 2.35 x 2.35 |
| 2 | Iliac wing right | IceRod | none | 2 min | 2 min | 2 min | 12,0 | 4,75 x 2,15 x 2,25 |
| 2 | Iliac wing left | IceRod | none | 4 min | 2 min | 4 min | 12,0 | 4,35 x 2.25 x 2.35 |
| 3 | L2 | IceSphere | none | 6 min | 2 min | 6 min | 8,4 | 3,55 x 2,2 x 2,05 |
| 3 | L3 | IceSphere | none | 4 min | 2 min | 4 min | 10,6 | 3,8 x 2,3 x 2,3 |
| 3 | L4 | IceSphere | none | 6 min | 2 min | 8 min | 7,0 | 3,15 x 2,1 x 2 |
| 3 | L5 | IceRod | none | 2 min | 2 min | 2 min | 14,8 | 4,5 x 2,55 x 2,45 |
| 3 | Iliac wing right | IceRod | none | 2 min | 2 min | 2 min | 9,3 | 4,8 x 2 x 1,85 |
| 3 | Iliac wing left | IceRod | none | 4 min | 2 min | 4 min | 14,8 | 4,85 x 2,2 x 2,65 |

**Supplementary materials**

**Table 1**

Location, needle type used, time to visualization of the ice ball in the bone and soft tissue in the conventional and VNCa images, ice ball volume and dimension.
